# Extracellular Dopamine Potentiates Mn-Induced Oxidative Stress, Lifespan Reduction, and Dopaminergic Neurodegeneration in a BLI-3–Dependent Manner in *Caenorhabditis elegans*

**DOI:** 10.1371/journal.pgen.1001084

**Published:** 2010-08-26

**Authors:** Alexandre Benedetto, Catherine Au, Daiana Silva Avila, Dejan Milatovic, Michael Aschner

**Affiliations:** 1 Department of Pediatrics, Vanderbilt University, Nashville, Tennessee, United States of America; 2 Children's Hospital, Vanderbilt University, Nashville, Tennessee, United States of America; 3 London Centre for Nanotechnology, University College London, London, United Kingdom; 4 Center for Molecular Toxicology, Vanderbilt University, Nashville, Tennessee, United States of America; University of California San Francisco, United States of America

## Abstract

Parkinson's disease (PD)-mimicking drugs and pesticides, and more recently PD-associated gene mutations, have been studied in cell cultures and mammalian models to decipher the molecular basis of PD. Thus far, a dozen of genes have been identified that are responsible for inherited PD. However they only account for about 8% of PD cases, most of the cases likely involving environmental contributions. Environmental manganese (Mn) exposure represents an established risk factor for PD occurrence, and both PD and Mn-intoxicated patients display a characteristic extrapyramidal syndrome primarily involving dopaminergic (DAergic) neurodegeneration with shared common molecular mechanisms. To better understand the specificity of DAergic neurodegeneration, we studied Mn toxicity *in vivo* in *Caenorhabditis elegans*. Combining genetics and biochemical assays, we established that extracellular, and not intracellular, dopamine (DA) is responsible for Mn-induced DAergic neurodegeneration and that this process (1) requires functional DA-reuptake transporter (DAT-1) and (2) is associated with oxidative stress and lifespan reduction. Overexpression of the anti-oxidant transcription factor, SKN-1, affords protection against Mn toxicity, while the DA-dependency of Mn toxicity requires the NADPH dual-oxidase BLI-3. These results suggest that *in vivo* BLI-3 activity promotes the conversion of extracellular DA into toxic reactive species, which, in turn, can be taken up by DAT-1 in DAergic neurons, thus leading to oxidative stress and cell degeneration.

## Author Summary

In Parkinson's disease (PD), motor neurons that produce dopamine degenerate, leading to a characteristic syndrome including tremor, rigidity, and bradykinesia. The mechanisms leading to PD have been under intense investigation, identifying hereditary mutations responsible for about 8% of the cases. However, multiple environmental factors contribute to PD; and, amongst those, manganese (Mn) exposure from pesticides, industrial fumes, and gasoline additives has been robustly associated with PD. To gain insights into processes leading to the specific degeneration of dopaminergic neurons, we used a simple animal model, the nematode *Caenorhabditis elegans*, which, upon Mn exposure, recapitulates key molecular processes known to be involved in PD. Combining biochemistry and genetics, we demonstrate that dopamine secreted by the neurons and not intracellular dopamine is directly involved in the generation of toxic reactive oxygen species. We identify two essential mediators of this dopamine-dependent effect which are an extracellularly active enzyme called dual-oxidase and the dopamine re-uptake transporter. We also reveal that a transcription factor which is strongly expressed in two neurons involved in the regulation of aging is a powerful modulator of the dopamine-dependent toxicity. Our study establishes novel evidence of the link among PD, aging, and oxidative stress within the context of exposure to Mn.

## Introduction

Mn is the twelfth most prevalent natural element in the Earth's crust [1] and is an essential transition metal required for normal growth, development and cellular homeostasis [2], [3]. It acts as a cofactor for multiple enzymes (Mn superoxide dismutase, pyruvate carboxylase, arginase, and glutamine synthase) [4], [5], [6], [7], [8] and can substitute for magnesium (Mg) in many enzymatic reactions catalyzed by kinases. Although dietary Mn intake by the gastrointestinal tract (GIT) and excretion via the bile are tightly regulated [9], inhalation of toxic concentrations of Mn can lead to nasal and pulmonary inflammation, renal dysfunction and neurodegeneration [7], [10], [11], [12]. A recent study also suggests that high levels of Mn in drinking water (>300 µg/liter) are associated with reduced intellectual function in children [13]. Mn mining, steel manufacturing and welding represent occupational exposures linked to increased risk for parkinsonian syndrome [14], . In addition, Mn is used in other industrial and agricultural applications. Fungicides, such as Maneb or Mancozeb, increase the risk of environmental Mn exposure in agricultural workers [16]. An organic Mn compound, methylcyclopentadienyl Mn tricarbonyl (MMT), used as an octane booster or anti-knock agent in gasoline, has also been shown to cause adverse health effects [17], [18], [19], [20].

Exposure to high levels of Mn in occupational or environmental settings or disease conditions (hepatic encephalopathy) [21] is accompanied by Mn accumulation in specific brain regions that are highly sensitive to oxidative injury, namely the *substantia nigra* (SN), *globus pallidus* (GP) and *striatum* [22]. Excessive Mn deposition in these regions leads to dopaminergic (DAergic) neuronal loss accompanied by an extrapyramidal syndrome referred to as manganism. Manganism patients exhibit rigidity, tremor, dystonic movements and bradykinesia, all of which are also characteristic features of Parkinson's disease (PD) [23], [24], [25]. Exposure to Mn also represents a risk factor for PD [26], [27], [28]. Indeed, the strongest correlation between any type of environmental exposure and PD is noted in Mn-exposed human cohorts [29], and occupational exposure to Mn for >20 years or combined long-term exposures to Mn and Al (>30 years) are associated with an increased occurrence of PD [30]. Parkinsonism in welders (*vs.* non-welders) is clinically distinguishable only by age of onset (46 *vs.* 63 years, respectively) [28], and the prevalence of PD is higher among welders as compared with age-standardized individuals in the general population [31].

Appraisal of the literature strongly suggests that in addition to targeting similar brain areas and causing similar clinical syndromes, DAergic neurodegeneration associated with PD or PD-mimicking drugs (6-hydroxydopamine/6-OHDA, 1-methyl-4-phenylpyridium/MPP+, rotenone, paraquat) and Mn neurotoxicity share multiple common effector mechanisms, namely mitochondrial dysfunction, ATP depletion, aberrant signal transduction, oxidative stress, protein aggregation and the activation of cell death pathways [32]. Damage to DAergic nigral neurons that is induced by MPP+ and rotenone involves oxidative stress [33]. Oxidative damage also plays a significant role in 6-OHDA-induced DAergic neuronal cell death. In cell cultures, hydrogen peroxide (H_2_O_2_), superoxide ions and hydroxyl radicals [34] generated by the non-enzymatic breakdown of 6-OHDA and the direct inhibition of complex-I activity, lead to lipid peroxidation, protein denaturation and a decrease in glutathione (GSH), all hallmark features of post-mortem PD [35], [36], [37].

Intrastriatal Mn injections result in the loss of DAergic neurons, a process in which oxidative stress plays a significant role [38], [39], [40], resembling toxicity caused by the mitochondrial poisons, aminooxyacetic acid and MPP^+^ [41]. Similarly to mitochondrial inhibitors such as MPP^+^ [42], Mn increases *in vivo* synaptic glutamate concentrations, which leads to excitotoxic and oxidative injury [43] and interfers with ATP synthesis [44]. Analogous to MPP^+^ and 6-OHDA, Mn elevates intracellular H_2_O_2_ and related peroxides [45] and reduces tyrosine hydroxylase (TH) activity and intracellular antioxidant levels (GSH, thiols, catalase) in DAergic neurons [43], [46], [47], [48]. Intracellular Mn^2+^ inhibits the mitochondrial complex-I, a feature inherent to PD and its experimental models (MPP^+^, 6-OHDA, rotenone, paraquat) [43]. A link between mitochondrial impairment, oxidative stress and increased α-synuclein aggregation is well documented for Mn and in various models of PD [37], [43], [49], [50], [51]. Studies have also confirmed that treatment with Mn in a pre-parkinsonian state (6-OHDA) significantly exacerbates neurobehavioral impairment in the rat, not only suggesting that Mn exposure may increase the risk of injury in subpopulations that are in a pre-parkinsonism state, but also pointing to the convergence of signaling pathways that lead to such injury [52].

MPP^+^ and 6-OHDA exposures as well as wild-type or mutant α-synuclein overexpression cause specific DAergic neurodegeneration in the worm [53], , a process which involves ATP depletion and oxidative stress [36], [57], [58], analogous to vertebrate models of PD. It was further confirmed that *C. elegans* orthologues of PD-associated genes play a role in α-synuclein toxicity and DAergic neurodegeneration. Additionally, conserved genetic networks were identified in *C. elegans* that potentiate or protect against α-synuclein toxicity, such as the torsin pathway [36], [59], [60], [61], [62], [63], [64]. A link between α-synuclein and Mn toxicity was also demonstrated in the worm [59], [65]. Furthermore, we previously established that Mn uptake and toxicity pathways in *C. elegans* relate to those described in vertebrates, which involves the NRAMP/DMT family of metal transporters and leads to defects in the developmental and excretory systems [66]. Here we show that at a sub-lethal range of concentrations, acute Mn exposure leads to a specific and dose-dependent neurodegeneration of all *C. elegans* DAergic neurons, while sparing other neurotransmitter systems, findings that corroborate the specificity of DAergic sensitivity shared with vertebrate models. We investigated the causes of this DAergic specificity, and we demonstrated that endogenous extracellular, but not intracellular DA potentiates Mn toxicity and that Mn-induced neurodegeneration requires the DAergic neuron-specific dopamine re-uptake transporter, DAT-1. We also found that Mn toxicity in the worm is associated with increased reactive-oxygen species (ROS), lipid peroxidation and lifespan reduction, all of which were dependent on extracellular DA concentrations. Additionally, we observed a relocation of the oxidant-responsive transcription factor, SKN-1, in ASI nuclei upon Mn exposure, whereas SKN-1 overexpression afforded protection against Mn-induced toxicity. Finally, we identified the NADPH dual-oxidase, BLI-3, as a key mediator of the DA-dependency of Mn toxicity, suggesting that BLI-3 potentiates the formation of ROS from DA-derived species obtained through the reaction of divalent Mn and extracellular DA.

## Results

### *C. elegans* DAergic neurons degenerate in a specific and concentration-dependent manner upon exposure to Mn

**Figure 1:**
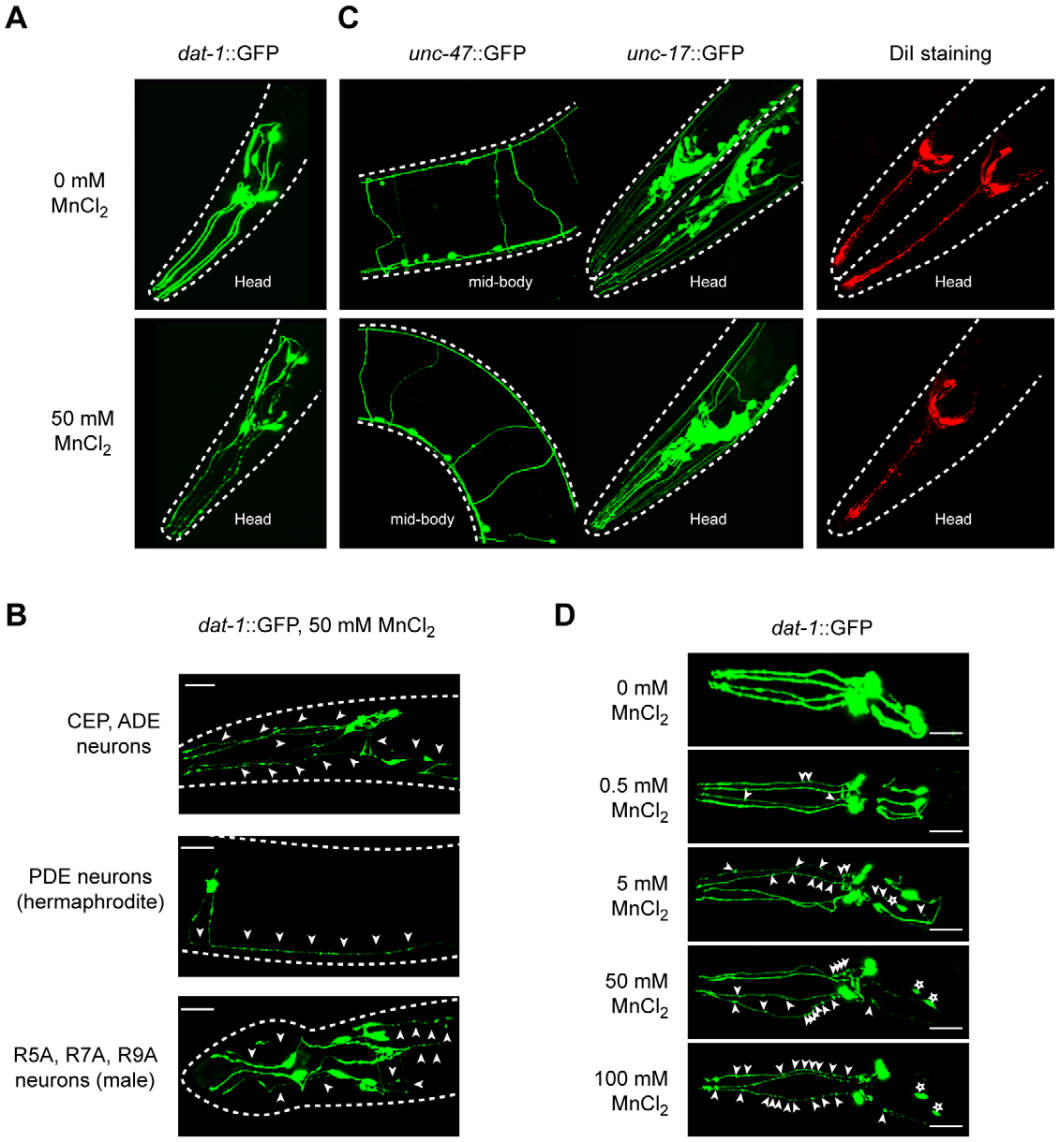
Dopaminergic neurons specifically degenerate in a dose-dependent manner upon Mn acute exposure. (A) Mechanosensory termini of the CEP and ADE dopaminergic neurons in *dat-1::*GFP expressing worms display discontinuous GFP staining 24 h after 50 mM Mn acute exposure at L1, revealing their degeneration. (B) Adult CEP, ADE, PDE and male specific R5A, R7A and R9A pairs of neurons are also affected following 50 mM MnCl_2_ acute exposure at L1. (C) GABAergic (*unc-47::*GFP), cholinergic (*unc-17::*GFP) and chemosensory neurons (DiI) are not affected by Mn exposure at concentrations inducing dopaminergic neurodegeneration (50 mM here). (D) CEP and ADE degeneration upon Mn exposure is dependent on Mn concentration. White arrowheads indicate neuronal processes exhibiting abnormal discontinuous GFP signal. White stars indicate the position of degenerated neuron cell bodies.

**Figure 2:**
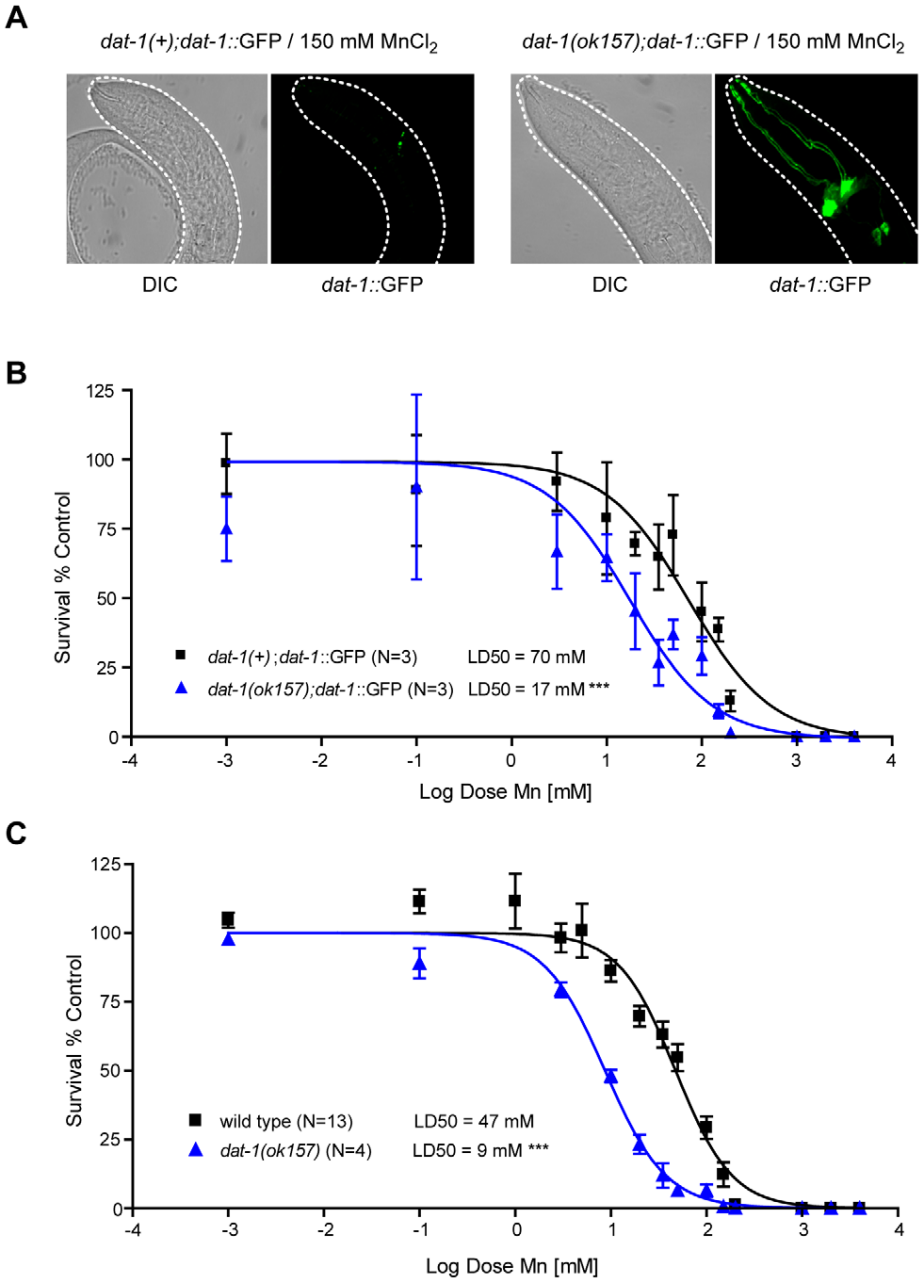
*dat-1* loss of function induces an overall hypersensitivity to Mn toxicity, but confers neuroprotection to the dopaminergic system. (A) Most surviving *dat-1::*GFP expressing *dat-1(ok157)* mutants fail to exhibit dopaminergic neurodegeneration, while surviving *dat-1::*GFP expressing wild-type worms always display dopaminergic processes loss and cell body shrinkage upon 150 mM Mn acute exposure. (B) *dat-1::*GFP expressing *dat-1(ok157)* mutants are hypersensitive (p<0.001) to Mn exposure, with a LD_50_ = 17 mM. C) *dat-1(ok157)* is hypersensitive (p<0.001) to Mn-induced lethality, with a LD_50_ = 9 mM.

We first ascertained the suitability of Mn-exposed *C. elegans* as an *in vivo* model for manganism and PD and examined whether features inherent to mammalian DAergic neurodegeneration can be convincingly recapitulated in this model. We took advantage of the BY200 strain, which expresses the green fluorescent protein (GFP) under the control of the DAergic-specific dopamine re-uptake transporter 1 promoter, *dat-1::GFP(vtIs1)* (Figure 1A). After acute exposure to Mn, a dose-dependent neurodegeneration was observed in all DAergic neurons, namely the 4 CEP, 2 ADE, 2 PDE and the male specific R5A, R7A and R9A pairs of neurons (Figure 1A and 1B). Typically, the primary defects were observed in neuron extensions, such as CEP mechanosensory processes, resulting in discontinued and punctuated GFP labeling (Figure 1A, 1B, 1D, arrowheads). With an increased dose (Figure 1D, upper to lower panels) or longer exposure to Mn (data not shown), these defects were exacerbated, leading to shortening or disappearance of the neuronal extensions (Figure 1D, lower panels) and eventually neuronal death as revealed by the shrinkage of the cell body, and ultimately, complete loss of GFP (data not shown, Figure 2A left panel). This effect was specific to DAergic neurons, since it was not observed in GABAergic, cholinergic, glutamatergic (Figure 1C) or in other biogenic-amine systems (data not shown). Despite the direct exposure of chemosensory neurons to the Mn-containing solution, DiI staining in these neurons failed to reveal any degeneration (Figure 1C), confirming the specificity of the Mn-induced DAergic neurodegeneration.

### DAergic neurodegeneration requires the DA re-uptake transporter DAT-1

The selectivity of Mn-induced neurodegeneration to DAergic neurons suggests that some factor(s) specific to these neurons sensitize(s) them to Mn-induced toxicity. To determine which factor(s) may account for this effect, a candidate gene approach was employed, starting with a gene known to be specific to the DAergic neurons, namely, *dat-1*. DAT-1 is the *C. elegans* orthologue of the vertebrate DAT, which is a highly conserved member of a family of transporters involved in neurotransmitter clearance, including the GABA re-uptake transporters, the GATs, the serotonin re-uptake transporter, SERT, and the excitatory amino-acid transporters, the EAATs. DAT is specifically responsible for DA clearance at the synapse, removing excessive extracellular DA into presynaptic DAergic termini [67], [68], [69]. Accordingly, chemical inhibition of DAT or *DAT* loss-of-function leads to high extracellular DA levels [70], [71]. In *C. elegans*, the *dat-1(ok157)* loss-of-function mutant displays a swimming-induced paralysis (SWIP) phenotype, likely due to the hyper-activation of DA-responsive motorneurons exposed to excessive synaptic DA concentrations [71]. *C. elegans dat-1* is also required for 6-OHDA DAergic neuron toxicity as noted in *dat-1::GFP* transgenic worms [56]. Given the specific neurodegeneration of *dat-1::GFP*-expressing neurons upon 6-OHDA or Mn exposure, we hypothesized that *dat-1* is required for Mn-induced DAergic degeneration. Accordingly, *dat-1(ok157)* worms expressing *dat-1::GFP* were exposed to graded Mn doses in parallel with wild-type worms expressing the same *dat-1::GFP* array, and both strains were scored for DAergic neuron defects. Mn treatment was associated with marked DAergic neurodegeneration in wild-type worms, while *dat-1* mutant DAergic neurons were not significantly affected even at the highest doses of Mn (Figure 2A) exposure for which fewer than 5% of the worms survived (Figure 2B). Accordingly, *dat-1* mutant worms exposed to Mn most likely died from osmoregulation defects before displaying any neurodegeneration in DAergic neurons. These results established that DAergic neurodegeneration in Mn-exposed worms requires a functional DAT-1 transporter.

### *dat-1(ok157)* mutant worms are hypersensitive to Mn-induced toxicity

Additional studies showed that the protective effect of *dat-1* loss-of-function on DAergic neurodegeneration was not due to an indirect effect, such as an adaptive mechanism resulting in reduced Mn-uptake. First, the lethal dose 50 at 24 h (LD_50_, dose of Mn exposure at which 50% of the animals die from the treatment) for both transgenic strains, *dat-1(ok157)*; *dat-1::GFP* and *dat-1(+)*; *dat-1::GFP*, was determined. Notably, the *dat-1(ok157)*; *dat-1::GFP* strain showed significant hypersensitivity (p<0.001) to Mn-induced toxicity with a LD_50_ = 20 mM, whereas the *dat-1(+)*; *dat-1::GFP* strain exhibited a LD_50_ = 74 mM (Figure 2B). Next, the non-transgenic *dat-1(ok157)* and the N2 wild-type strains were treated with the same range of Mn doses (0.001 mM to 1 M). Both non-transgenic strains were slightly more sensitive than the corresponding *dat-1::GFP*-expressing strains (Figure 2C). This “protective effect” of the transgene expression was not specific to the *dat-1::GFP* construct and was systematically observed with any type of transcriptional GFP-expressing construct (data not shown). Transgenic worms may experience higher basal levels of stress associated with increased stress-response protein levels, enabling them to better cope with environmental stress. Nevertheless, *dat-1(ok157)* mutants exhibited hypersensitivity to Mn-induced lethality (p<0.001), which was characterized by a LD_50_ = 9 mM as compared to a LD_50_ = 47 mM for wild-type worms (Figure 2C). This observation rules out the possibility that *dat-1(ok157)* takes up less Mn. Moreover, it shows that upon Mn exposure, DAT-1 loss-of-function is detrimental to worm survival, though protective to DAergic neurons.

### Sub-lethal exogenous DA pre-treatment potentiates Mn toxicity

**Figure 3:**
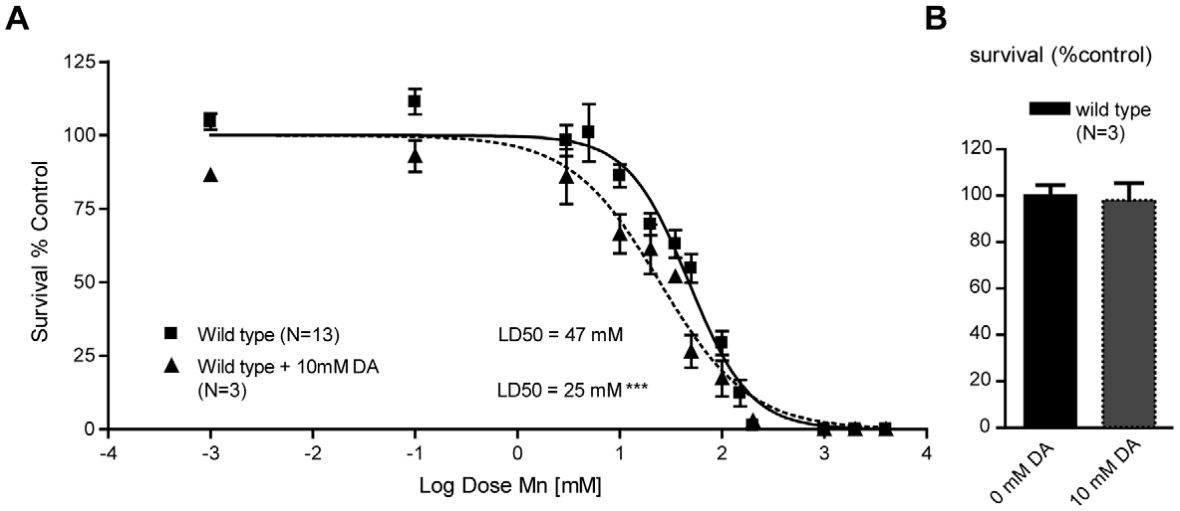
Sub-lethal dopamine pre-treatment sensitizes wild-type worms to Mn exposure. (A) Pre-treatment with 10 mM dopamine renders wild-type worms hypersensitive (p<0.001) to subsequent MnCl_2_ exposure, bringing the LD_50_ from 47 mM to 25 mM. (B) 10 mM L1 acute treatment does not affect wild-type worm survival at 24 h.

The importance of a functional DAT-1 in conferring selective neurodegeneration in *C. elegans* DAergic neurons upon acute Mn exposure reflected upon earlier work with the PD-mimicking drug and DA analogue, 6-OHDA. Indeed, 6-OHDA induced a dose-dependent DAergic neurodegeneration in *C. elegans*, which was prevented by DAT-1 mutations [56], [72]. The fact that both Mn and 6-OHDA induce neurotoxicity through DAT-1 raises a question regarding the relationship between Mn and DA analogues upstream of DAT-1 in the DAergic neurodegeneration pathway. It has been hypothesized that Mn, in its divalent or trivalent cationic form, like iron, reacts with biogenic amines, such as DA, to generate ROS [73], [74], [75]. It is also known that DA and its derivatives, such as L-DOPA and 6-OHDA, can be toxic to the mammalian DAergic system and can lead to intracellular oxidative stress alone or in combination with metals and ensuing DAergic neurodegeneration [46], [76], [77]. Accordingly, we hypothesized that DA and Mn have a synergistic toxic effect in the worm. To test this hypothesis, worms were acutely treated with 10 mM DA prior to Mn exposure. DA pre-treatment led to a significant leftward shift in the Mn dose-response survival curve (p<0.001) with a LD_50_ = 25 mM (Figure 3A), while 10 mM acute DA treatment alone did not affect the worms' survival (Figure 3B). These results establish that exogenously applied DA and Mn can act synergistically *in vivo* to promote increased toxicity in the worm.

### Endogenous DA potentiates Mn toxicity

**Figure 4:**
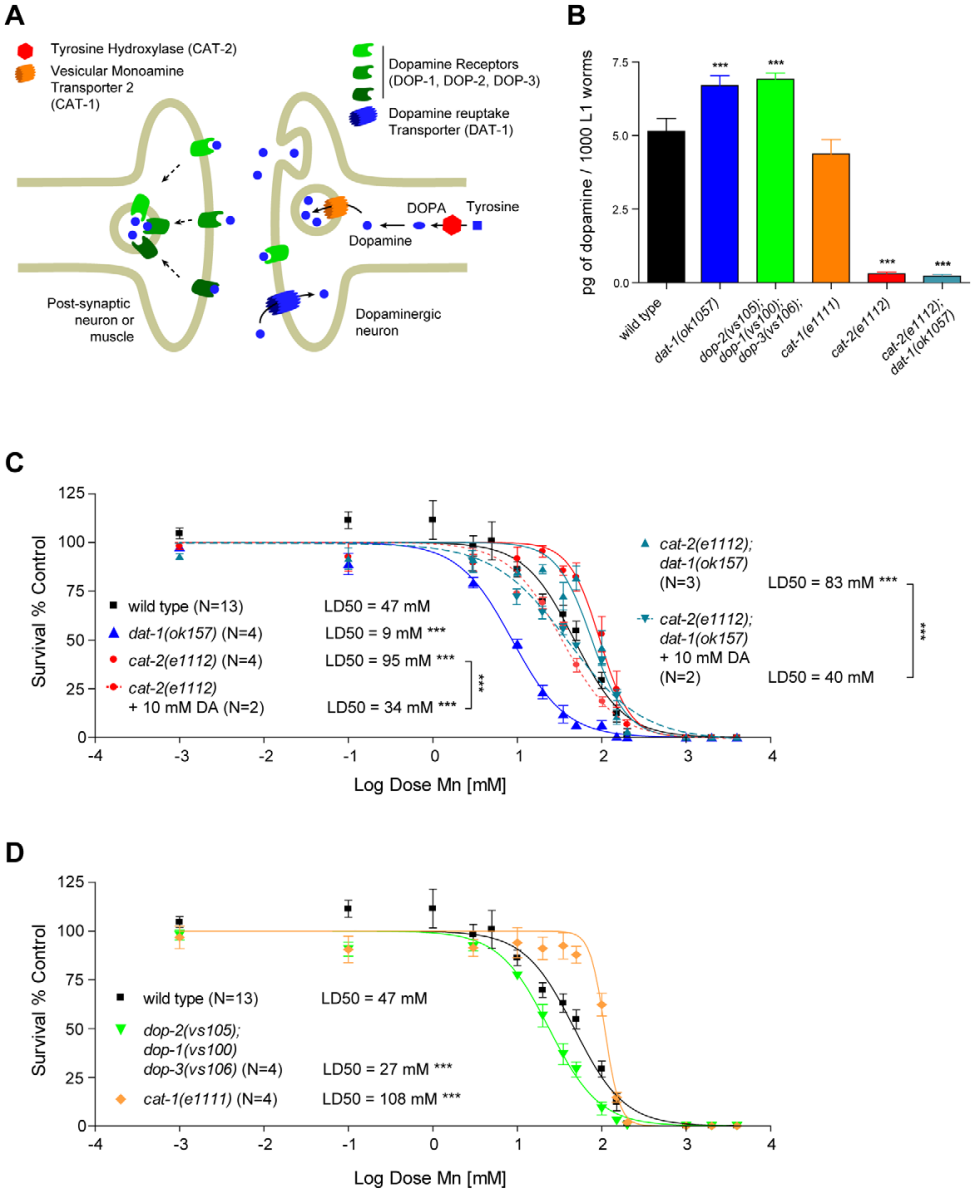
Increased extracellular, but not intracellular, dopamine levels confer hypersensitivity to Mn toxicity while decreased levels are protective. (A) Schematic representation of a generic dopaminergic synapse in *C. elegans*. (B) Total DA levels in selected mutants relevant to the study at the L1 larval stage. *dat-1(ok157)*, *dop-2(vs105);dop-1(vs100) dop-3(vs106)* display significantly (p<0.001) higher dopamine levels than WT or other mutants, while dopamine measurement in *cat-2(e1112)* and *dat-1(ok157);cat-2(e1112)* did not reach a significant value compared to the background levels measured in our experiment. (C) *dat-1(ok157)* is hypersensitive (p<0.001) to Mn-induced lethality, with a LD_50_ = 9 mM. *cat-2(e1112)* is hyper-resistant (p<0.001) to Mn-induced lethality, with a LD_50_ = 95 mM. *cat-2(e1112)* rescues *dat-1(ok157)* hypersensitivity in the double-mutant *cat-2(e1112)*; *dat-1(ok157)*, which LD_50_ = 83 mM is not significantly different from *cat-2(e1112)* mutant. 10 mM DA pre-treatment restores Mn sensitivity (p<0.001) in both *cat-2(e1112)* and *cat-2(e1112);dat-1(ok157)*, with respective LD_50_ of 34 mM and 40 mM. (D) The VMAT2 mutant *cat-1(e1111)* exhibits hyper-resistance to Mn (p<0.001), with a LD_50_ = 108 mM; while the DA receptor mutant *dop-2(vs105);dop-1(vs100) dop-3(vs106)* is hypersensitive to Mn exposure (p<0.001), with a LD_50_ = 27 mM.

In certain mutant backgrounds, such as vertebrate *DAT* mutants, DA levels were reported to be abnormally elevated due to a lack of DA clearance at the DAergic synapses [71]. Therefore, the effect of DAT-1 loss-of-function on Mn sensitivity may reflect increased endogenous levels of DA (Figure 4A). To test this hypothesis, we measured DA levels in the *C. elegans dat-1(ok157)* mutants. We found that *dat-1(ok157)* endogenous DA levels were significantly higher when compared with wild-type worms (Figure 4B). To confirm the role of endogenous DA in Mn toxicity, we tested the effect of endogenous DA depletion on Mn sensitivity using the *cat-2(e1112)* loss-of-function mutant, in which the tyrosine hydroxylase (TH) activity is abolished, resulting in the absence of DA synthesis (Figure 4A and 4B). *cat-2(e1112)* mutants exposed to Mn revealed significant hyper-resistance (p<0.001), with a LD_50_ = 95 mM (Figure 4C). Moreover, the *e1112* deletion rescued the *dat-1(ok157)* hypersensitivity (LD_50_ = 9 mM, Figure 3A) and led to hyper-resistance to Mn (p<0.001) with a LD_50_ = 83 mM for *cat-2(e1112);dat-1(ok157)* worms (Figure 4C). The LD_50_ difference between *cat-2(e1112)* and *cat-2(e1112);dat-1(ok157)* worm strains was not statistically significant (p>0.05), suggesting that the *cat-2(e1112)* effect takes place upstream of *dat-1(ok157)* in the same pathway (Figure 4A). Finally, we supplemented *cat-2(e1112)* mutants with 10 mM DA exposure prior Mn treatment, which rendered those mutants hypersensitive (p<0.001) to Mn with a LD_50_ = 34 mM (Figure 4C). Pre-exposure to 10 mM DA of *cat-2(e1112);dat-1(ok157)* worms led to a comparable LD_50_ = 40 mM, not significantly different from *cat-2(e1112)* mutants pre-exposed to 10 mM DA, but significantly different from *cat-2(e1112);dat-1(ok157)* without DA pre-exposure (Figure 4C). These data further confirm that endogenous DA level regulation plays a key role in Mn toxicity and that DA itself is required for the full extent of Mn toxicity.

### Extracellular DA, and not intracellular DA, is involved in Mn-induced lethality

Next, we determined which step along the DAergic metabolic pathway contributes to the synergistic toxic effect of Mn. To address this issue, endogenous DA levels at the L1 stage were measured in a combination of deletion mutants (Figure 4A). First, we sought to determine if DA release was necessary for the DA-dependent Mn-induced lethality. Accordingly, *cat-1(e1111)* mutants in which the vesicular monoamine transporter 2 (VMAT2) is defective, were exposed to Mn (Figure 4B). This mutant is unable to pack DA in secretory vesicles, thus abolishing DA synaptic release, while still producing normal levels of DA, at least at the L1 stage (Figure 4A). Upon exposure to Mn, *cat-1(e1111)* mutants were more resistant to Mn-induced lethality than wild-type worms (p<0.001), with a LD_50_ = 108 mM (Figure 4D), which was statistically insignificant when compared to the *cat-2(e1112)* mutant LD_50_ (p>0.05). These data establish that synaptic DA release is necessary for DA-dependent Mn-induced lethality and that extracellular DA, and not presynaptic DA, mediates this effect.

Furthermore, the data imply that blockage of extracellular DA receptor activity (and the ensuing increase in extracellular DA levels) should exacerbate Mn sensitivity. *C. elegans* expresses three DA receptors, namely DOP-1, DOP-2 and DOP-3 (Figure 4A). A triple knock-out was generated [78], and as expected, the *dop-2(vs105)*; *dop-1(vs100) dop-3(vs106)* mutant exhibited higher levels of DA (Figure 4B) and was hypersensitive (p<0.001 vs. wild-type) to Mn-induced lethality with a LD_50_ = 27 mM (Figure 4D). However, this mutant was not as sensitive to Mn as the *dat-1(ok157)* mutant, probably because the DA clearance activity of the wild-type DAT-1 limited the extent of the extracellular DA accumulation in the *dop-2(vs105)*; *dop-1(vs100) dop-3(vs106)* triple mutant. These results confirm that extracellular, and not intracellular DA is responsible for the DA-dependent Mn-induced lethality.

### DA-dependent Mn toxicity is associated with decreased longevity

**Figure 5:**
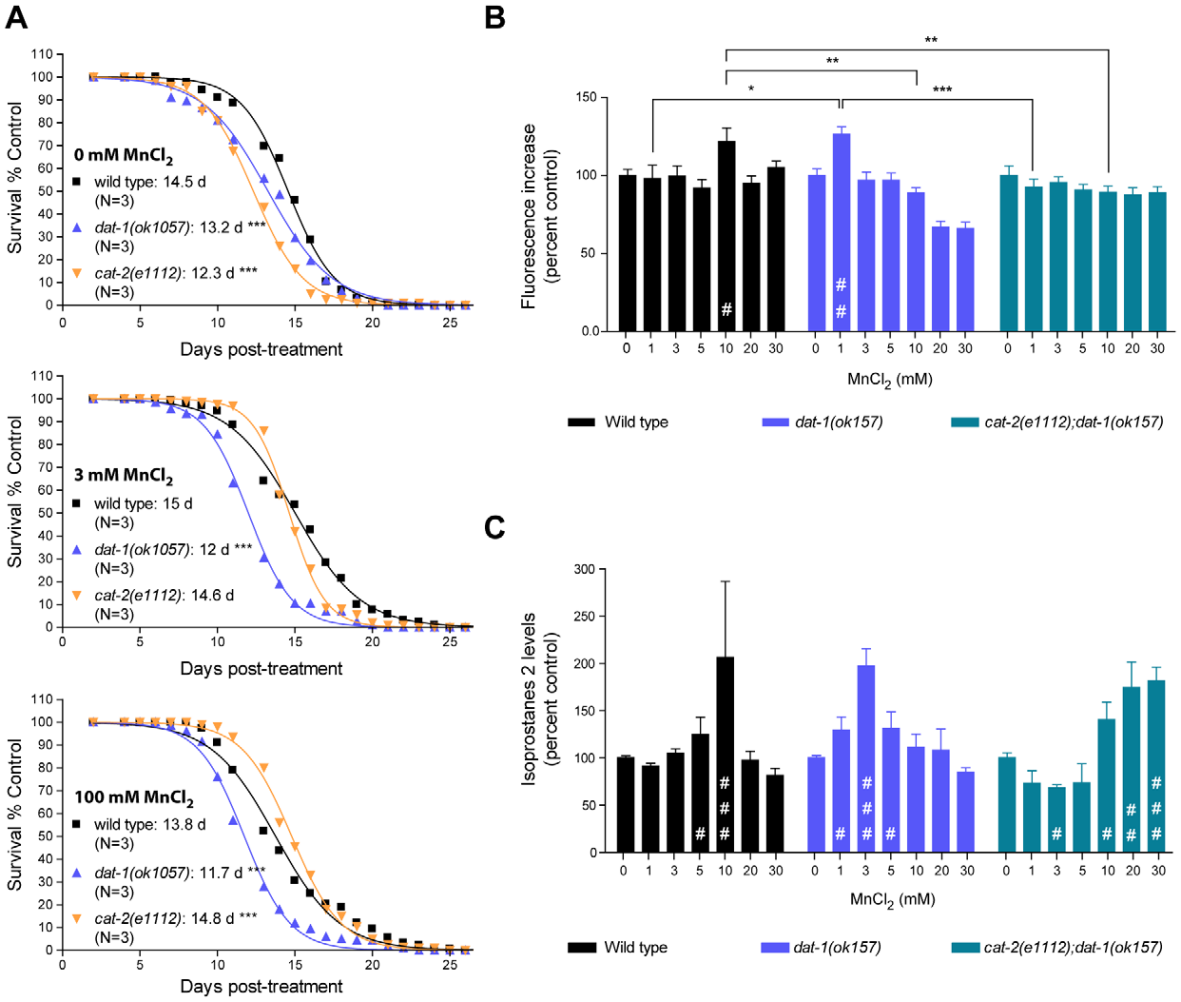
Dopamine-dependent Mn toxicity is associated with decreased lifespan and increased oxidative stress. (A) *cat-2(e1112)* and *dat-1(ok1057)* mutants both show a reduced lifespan (p<0.001) upon Mn acute treatment. *dat-1(ok1057)* exhibit a further reduced lifespan, whereas *cat-2(e1112)* show a significant improvement upon 3 mM MnCl_2_ acute treatment and a further increased lifespan (p<0.001) upon 100 mM MnCl_2_ acute treatment. Plotted values represent averages of three independent experiments and the curves represent the best sigmoidal fit. (B) Wild-type worms exhibit significantly higher reactive oxygen species (ROS) levels upon 10 mM Mn treatment (p<0.05), while *dat-1(ok157)* mutant show a significant increase after only 1 mM Mn exposure (p<0.01), and *cat-2(e1112);dat-1(ok157)* display normal ROS levels up to 30 mM Mn exposure. (C) Wild-type worms display significantly increased isoprostane-2 (F2IP) levels upon 10 mM Mn treatment (p<0.001), while *dat-1(ok157)* mutant show a significant increase after 3 mM Mn exposure (p<0.001), and *cat-2(e1112);dat-1(ok157)* upon 20 mM (p<0.05) and 30 mM (p<0.01).

To further investigate the pathways involved in the DA-dependent Mn toxicity, and especially its long-term effects, we scored the survival rate of worms which survived acute Mn-exposure. For each strain tested, we picked healthy-looking young adults homogeneous in stage at 48 h post-exposure (for all strains tested these animals were representative of more than 90% of the surviving population), and disregarded sick-looking or developmentally delayed worms. Accordingly, all animals selected survived at least 4 days in adulthood, regardless of their genetic background. This bias was deemed necessary to ensure that any significant differences potentially observed between strains could not be attributed to early toxicity events. In accordance with previous reports [79], low doses of Mn (3 mM for 30 min) led to a minor (albeit insignificant) increase in the surviving wild-type lifespan from 14.5 days post-treatment (dpt) to 15 dpt (Figure 5A, upper and middle graphs). Higher doses of Mn (100 mM for 30 min) led to a decrease in lifespan from 14.5 dpt to 13.8 dpt (p<0.001, Figure 5A, bottom graph). *dat-1(ok1057)* mutants exhibited a significantly shorter lifespan compared with wild-type worms in the absence of Mn treatment (13.2 dpt, p<0.001), which was further reduced (p<0.001) by both low- and high-dose acute Mn treatments (12 dpt and 11.7 dpt, respectively). Conversely, the lifespan of short-lived *cat-2(e1112)* mutants (12.3 dpt in absence of Mn, p<0.001) was significantly increased with increased Mn dosing (p<0.001, 14.6 dpt upon 3 mM and 14.8 dpt upon 100 mM acute Mn exposure). Moreover, the lifespan of the *cat-2(e1112)* mutants was significantly extended as compared to wild-type worms upon 100 mM Mn exposure (14.8 dpt vs 13.8 dpt, p<0.001), reaching untreated wild-type worm average lifespan (14.8 dpt *vs* 14.5 dpt). These observations made on worms, all of which survived for at least 5 days post-treatment, mirror the results obtained for lethality rates at 24 h post-treatment, indicating that the DA-dependent Mn toxicity observed upon acute Mn treatment has short- and long-term components, affecting both developmental (see also [66]) and aging processes.

### DA-dependent Mn-induced toxicity is associated with increased oxidative stress

Toxicity mechanisms implicated in neurodegenerative diseases, such as PD and Alzheimer's disease, involve oxidative stress [32], [35], [37], [80], [81], [82], [83]. In particular, DAergic cell loss in PD patients and experimental PD models invokes excessive ROS production [77], [84], [85], [86], [87], [88]. Moreover, oxidizing metals such as copper, Mn and iron in their 2+ or 3+ states are known to be sources of ROS via the Fenton reaction [89]. In *C. elegans*, pre-treatment with anti-oxidants such as ebselen, affords protection against acutely-induced Mn toxicity (Avila and Aschner, unpublished data). To determine if oxidative stress plays a role in DA-dependent Mn toxicity in *C. elegans*, a double-pronged approach was undertaken. First, the presence of ROS in response to acute Mn treatment was determined with the fluorescent dye, 2′7′ dichlorodihydrofluorescein diacetate (H2DCF-DA). As shown in Figure 5B, Mn-treated wild-type worms showed a significant (p<0.05) increase in fluorescence at sub-lethal Mn doses (10 mM). Interestingly, *dat-1(ok157)* mutants showed a significant increase in fluorescence (p<0.01) after 1 mM exposure, whereas *cat-2(e1112)*; *dat-1(ok157)* double mutants did not show any significant increase in fluorescence (p>0.05) upon exposure up to 30 mM Mn (Figure 5B). To confirm these results, we also used a more quantitative method involving the measurement of lipid peroxidation. Isoprostanes F2 (F2IP) and F3 (F3IP) are oxidation products of arachidonic acid (AA), which is released from membranes upon oxidative injury [90], [91], [92]. A new protocol was developed to extract and measure F2IP and F3IP from *C. elegans*. Corroborating the H2DCF-DA results (Figure 5B), *dat-1(ok157)* mutants exhibited significantly higher levels of F2IP (Figure 5C) and F3IP (data not shown) upon 1, 3 and 5 mM Mn exposure (p<0.05, p<0.001, p<0.05, respectively), as compared to wild-type worms, which showed a significant increase in F2IP content upon 5 and 10 mM Mn exposure (p<0.05 and p<0.001, respectively) (Figure 5C). The double mutant *cat-2(e1112);dat-1(ok157)* consistently displayed decreased F2IP levels between 1 and 5 mM Mn, with significantly lower levels at 3 mM (p<0.05), and exhibited significantly higher F2IP levels upon 10, 20 and 30 mM acute Mn exposure (p<0.05, p<0.01, p<0.001, respectively) (Figure 5C). Thus, to attain maximal levels of F2IP (corresponding to a 2-fold increase), higher doses of Mn were required in *cat-2(e1112);dat-1(ok157)* mutants compared to wild-type and in wild-type compared to *dat-1(ok157)*. Taken together, the H2DCF-DA and isoprostane measurements show that acute Mn treatments induce oxidative stress in *C. elegans*. In addition, the degree of oxidative stress depends on the extracellular DA content. These experiments strongly suggest that the DA-dependent Mn-induced toxicity involves oxidative stress.

### The anti-oxidant transcription factor, SKN-1, protects against Mn toxicity and relocalizes in amphide sensilla ASI neuron nuclei upon Mn exposure

**Figure 6:**
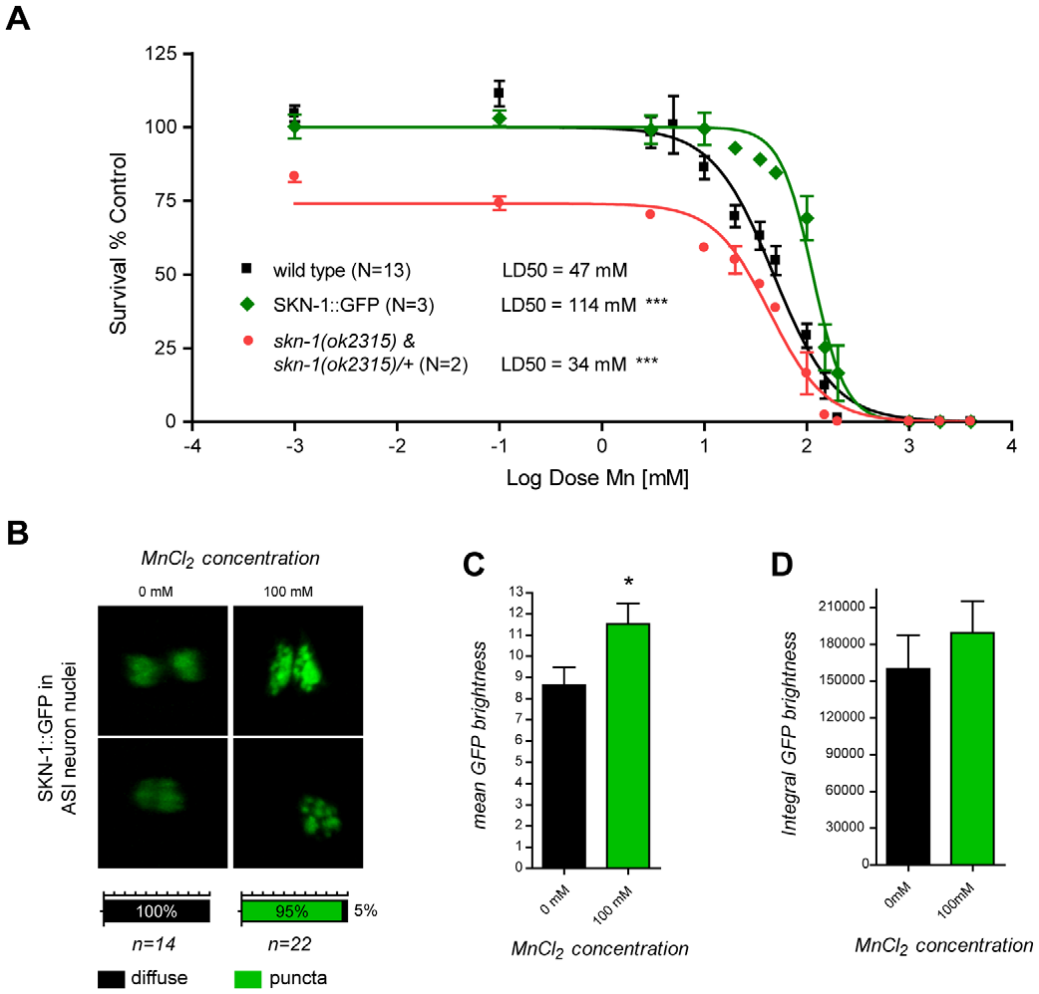
skn-1 mutants are hypersensitive while SKN-1::GFP overexpressing worms are hyper-resistant to Mn toxicity and respond to Mn exposure by relocalizing SKN-1::GFP to nuclear puncta in ASI neurons. (A) SKN-1::GFP expressing worms are hyper-resistant (p<0.001) to Mn-induced lethality, with a LD_50_ = 114 mM, while mixed *skn-1(ok2315)* and *skn-1(ok2315)/+* are hypersensitive (p<0.001) to Mn exposure, with a LD_50_ = 34 mM. (B) SKN-1::GFP nuclear localization in ASI neurons reorganizes in puncta 1 hour after MnCl_2_ exposure. (C) SKN-1::GFP mean brightness significantly increases (p<0.05) in ASI nuclei upon Mn exposure while integral brightness does not (D).

An alternative functional way to establish oxidative stress, especially in its earliest stages, is to demonstrate a physiological response to it. Sodium arsenite exposure in *C. elegans* has previously been shown to induce a strong intestinal expression of the anti-oxidant response gene, *skn-1*, the orthologue of the vertebrate gene, *Nrf2* [93]. SKN-1 initiates the development of the digestive system and feeding during the earliest embryonic stages, and post-embryonically, is required for a normal lifespan and adequate resistance to stress [94], [95], [96]. Consistent with these observations, *skn-1* deletions or loss-of-function mutations suppress oxidative stress resistance [97]. Aging in *C. elegans* is delayed when SKN-1 is transgenically expressed, and a mutant *skn-1* form that constitutively localizes to nuclei increases the worm's lifespan [94], [97], [98], [99]. To determine if SKN-1 can afford protection against Mn toxicity and to confirm that oxidative stress plays an important role in Mn-induced toxicity in *C. elegans*, SKN-1::GFP over-expressing worms were exposed to Mn. These worms exhibited a strong hyper-resistant phenotype to Mn exposure with a LD_50_ = 114 mM (Figure 6A). This effect was significantly greater in comparison to wild-type (p<0.001) or GFP expression alone (p<0.01) under various promoters (cf. *dat-1*::GFP, Figure 2; data not shown) despite the aforementioned protective effect of GFP expression, suggesting that SKN-1 directly protects against Mn toxicity. Moreover, a mixed population of two-third heterozygous and one-third homozygous deletion-mutant *skn-1(ok2315)* expressing a truncated SKN-1 protein, exhibited increased sensitivity to Mn exposure with a LD_50_ = 34 mM (Figure 6A), confirming that SKN-1 is required for mediating optimal resistance to Mn exposure. Unexpectedly, no obvious increase in SKN-1::GFP intestinal expression was noted upon Mn exposure, possibly because SKN-1 was already over-expressed or because it is not activated by Mn. However, a notable change in the SKN-1::GFP localization pattern in the ASI neuron was associated with Mn exposure, where SKN-1::GFP relocalized in discrete nuclear puncta, a result distinctly different from the diffuse pattern exhibited in non-Mn exposed worms (Figure 6B). This change was associated with a significantly increased average nuclear density of SKN-1::GFP (p<0.05, Figure 6C) but a non-significant increase in ASI expression, as revealed by the integral GFP intensity measurements (Figure 6D). These results support the notion that Mn exposure triggers an increase in ROS levels, thus activating the antioxidant response pathway, at least in ASI neurons. The data also indicate that the nuclear relocation of SKN-1, rather than its increased expression, is responsible for this protective effect against Mn toxicity.

### The dual-oxidase mutant *bli-3(e767)* is resistant to DA-dependent Mn toxicity

**Figure 7:**
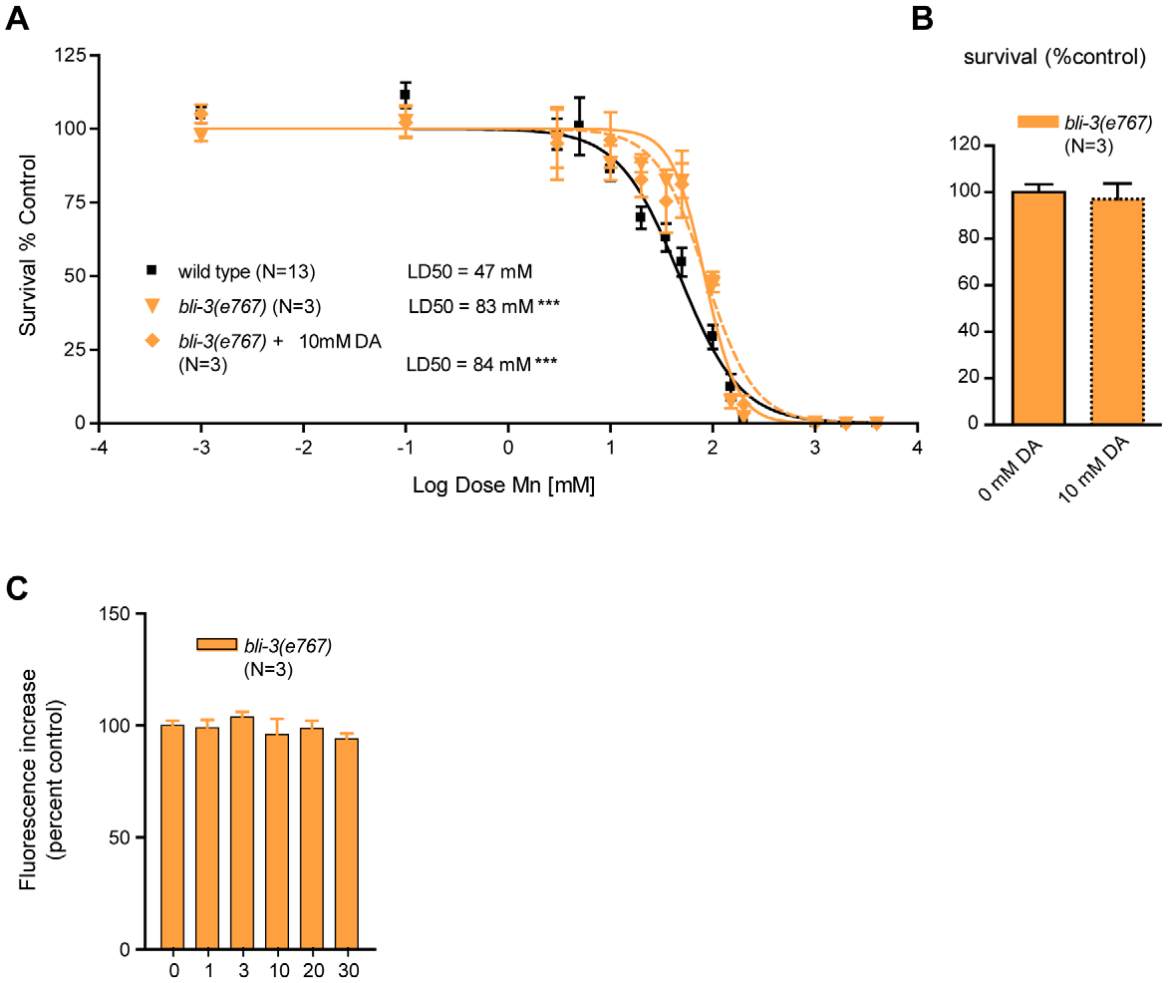
*bli-3* loss of function is hyper-resistant to Mn toxicity, insensitive to dopamine pre-treatment, and exhibits no dose-dependent Mn-associated ROS production. (A) *bli-3(e767)* is hyper-resistant (p<0.001) to Mn-induced lethality, with a LD_50_ = 83 mM. Pre-treatment with 10 mM dopamine (DA) does not change its hyper-resistance (p<0.001) and its LD_50_ = 84 mM is not significantly different from animals that were not pre-treated. (B) DA pre-treatment does not affect *bli-3(e767)* survival at 24 h. (C) Mn exposures from 1 mM up to 30 mM do not affect ROS levels significantly in *bli-3(e767)* worms (p>0.1). D) *bli-3(e767)* mutant does not show any significant difference in ROS levels upon Mn exposure up to 30 mM.

Given that Mn induces DA-dependent oxidative stress, we next sought to identify genetic factors involved in this process. In addition to the mitochondrial electron-transport chain (ETC), plasma membrane NADPH oxidases are major contributors to ROS production in rotenone, paraquat and MPP+-induced toxicity [86], [100], [101], [102], [103]. The *bli-3* gene encodes a *C. elegans* dual-oxidase orthologue to vertebrates DUOX1 and DUOX2, which is involved in the di-tyrosine bond formation in the worm cuticle [104] and pathogen-induced ROS production [105], [106]. Di-tyrosine bonds maintain cuticle integrity, and *bli-3(e767)* mutants display a blistered cuticle and a mild dumpy phenotype. This phenotype renders worms more sensitive to toxicants due to increased absorbance of the toxicants through the damaged cuticle [104]. Interestingly, *bli-3(e767)* worms displayed hyper-resistance to acute Mn treatments when compared to wild-type worms (p<0.001), with a LD_50_ = 83 mM (Figure 7A), suggesting that BLI-3 is involved in mediating Mn-toxicity, most likely by potentiating ROS production and oxidative stress. Notably, sub-lethal (Figure 7B) DA pre-treatment did not affect *bli-3(e767)* worm sensitivity to Mn: LD_50_ = 84mM (Figure 7A). Moreover, similar to *cat-2(e1112)* mutants (Figure 5B), *bli-3(e767)* mutants did not show any dose-dependent increase in ROS production from 1 mM to 30 mM Mn exposures (Figure 7C). ANOVA (comparing data from Figure 5B and Figure 7C) reveals that *bli-3(e767)* mutants show a significant difference in ROS production compared to *dat-1(ok157)* worms at 3 mM (p<0.001) and compared to wild-type worms at 10 mM (p<0.01), but no difference compared to *cat-2(e1112)* worms at any Mn concentration tested.

These observations strongly support the notion that BLI-3 is required for the ROS-associated potentiating effect of DA in Mn toxicity. Given the structural analogy between DA and tyrosine, DA or DA-derived species formed upon reaction with Mn may serve as substrates for BLI-3, facilitating their conversion to highly oxidized species, and in turn, potentiating oxidative stress.

## Discussion

**Figure 8:**
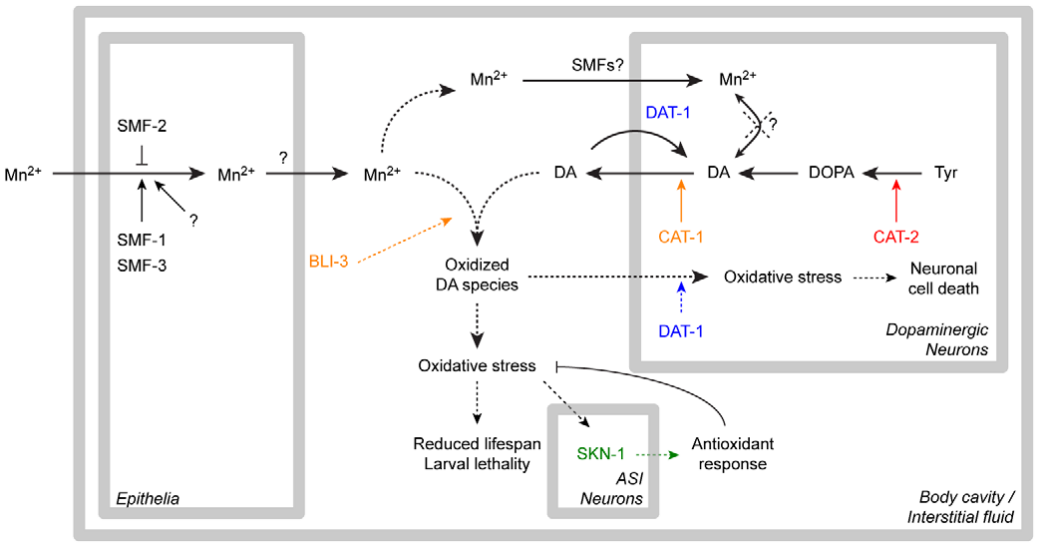
Recapitulative scheme. Manganese taken up by the epithelial DMT1-like SMF transporters is released in the body cavity (possibly by ferroportins), where it meets extracellular dopamine released from the dopaminergic synapses. The dual-oxidase BLI-3 is likely involved in the generation of oxidized dopamine-derived species, which can be taken up by DAT-1 leading to dopaminergic neurodegeneration, or are further increasing overall oxidative stress, associated with larval lethality and adult lifespan reduction. The ASI neurons in which SKN-1 nuclear relocation was observed could be involved in the orchestration of the overall antioxidant response. Dashed arrows highlight the pathways supported by this study. “SMFs?”: SMF transporters may be involved in direct Mn uptake in DAergic neurons according to earlier studies [65], [66].

This study establishes that Mn induces a specific and dose-dependent neurodegeneration of DAergic neurons in *C. elegans*, corroborating findings in rodents and non-human primates. This neurodegeneration was proven to require the DA re-uptake transporter, DAT-1, which is also the case in 6-OHDA-induced neurotoxicity. Combined, these observations suggest that upon Mn exposure, DAT-1 facilitates the intracellular transport of toxic species into DAergic neurons. Mn toxicity was associated with increased ROS generation and elevated F2IP levels as well as reduced longevity, all of which were aggravated by high endogenous DA levels and were attenuated by DA depletion. Consistent with a Mn-induced increase in oxidative stress, nuclear relocation of the anti-oxidant transcription factor, SKN-1, was noted in ASI neurons, and SKN-1 overexpression afforded protection against Mn-induced toxicity, while *skn-1* deletion rendered worms more vulnerable to Mn toxicity. Increased extracellular, but not intracellular DA levels resulting from direct DA exposure or genetic manipulation potentiated Mn-induced lethality, oxidative-stress and reduction in lifespan. Finally, our studies establish that the dual-oxidase, BLI-3, is involved in the DA-dependent Mn-induced toxicity and that *bli-3* loss-of-function suppresses the DA-dependency of Mn toxicity and associated oxidative stress. Combined, these data suggest that oxidation of extracellular DA promoted by the BLI-3 dual-oxidase activity triggers DA-derived ROS generation, which, in turn, are taken up into the DAergic terminals by DAT-1 and lead to specific DAergic neurodegeneration (Figure 8).

### Relevance of our findings to the study of manganism and PD in mammalian models

Mn-treated *C. elegans* recapitulate essential physiological aspects of parkinsonism, namely: the importance of NRAMP/DMT orthologues in the toxicity process [107], [108], [109]; the specificity and dose-dependency of the DAergic neurodegeneration [110], [111]; the involvement of DAT [112], [113], [114]; the synergy between DA and Mn [115]; and the associated oxidative stress [116], [117], [118].

As many divalent metallic cations are able to oxidize biogenic amines (amongst which DA and serotonin) *in vitro* via the Fenton's reaction, we carried out two control experiments to ascertain the specificity of the relationship between DA and Mn *in vivo*. Unlike *dat-1* mutation, deletion of the serotonin transporter (*mod-5* in *C. elegans*) did not affect Mn-induced lethality (LD_50_ = 45 mM, Figure S1), and DA-depleted *cat-2(e1112);dat-1(ok157)* mutants revealed greater susceptibility to iron-induced ROS production (p<0.05) than wild-type worms (Figure S2), supporting the fact that *in vivo*, metal-induced DA-dependent toxicity is specific to Mn. An additional novelty resides in the finding of the early and central role played by endogenous extracellular DA and the NADPH dual-oxidase in Mn-induced toxicity (Figure 4, Figure 5, and Figure 7). The centrality of extracellular DA in Mn-induced toxicity was further exemplified by the level of resistance to Mn conferred by mutations in *cat-1* (LD_50_ = 108 mM) and *cat-2* (LD_50_ = 95 mM), which approach those conferred by deletion of the Mn transporter, *smf-3* (LD_50_ = 126 mM) [66]. Moreover, Mn in its normal toxic range had a strong beneficial effect on the lifespan of DA-depleted *cat-2(e1112)* mutants (Figure 5A), further confirming that the DA-dependent component of Mn-induced toxicity accounts for a significant share of the overall toxicity. Importantly, the hypersensitivity to Mn of the DA receptor triple-knockout (Figure 4D), shows that Mn-induced neurodegeneration does not require DA receptors. Combined with the absence of post-synaptic neurodegeneration in *dop-1::GFP* and *dop-3::RFP*-expressing worms upon Mn exposure (data not shown) and despite the excitotoxic potential of other DA-related compounds, such as L-DOPA and 6-OHDA [119], the results establish that DA-dependent Mn toxicity does not involve excitotoxicity, in contrary to the glutamate-induced neurodegeneration involved in amyotrophic lateral sclerosis (ALS) [120]. Together, these results also provide a novel explanation for the requirement of DAT in the DAergic neurodegeneration *in vivo*. Rather than being responsible for cytosolic DA accumulation followed by downstream ROS production, which was considered as the reason for the sensitivity of DAergic neurons to PD-mimicking drugs (MPP^+^, 6-OHDA) [56], [114], [121], [122], DAT may facilitate the transport of extracellulary oxidized DA-derived species into DAergic neurons. In man and rat, subchronic Mn exposure has been shown to reduce DAT expression levels [123], [124]. Although it is unclear if it reflected a decline of DAergic neuron activity or a specific down-regulation of DAT, Mn interference with DAT activity should be further investigated. The importance of the NADPH dual-oxidase, BLI-3, in the DA-dependent Mn toxicity in *C. elegans* corroborates the up-regulation of its mammalian orthologue in PD patients and MPP^+^-exposed mice, as well as the protective effect conferred by its loss-of-function against oxidative stress and DAergic neurodegeneration in MPP^+^-exposed mutant mice [86]. Finally, DA-dependent Mn-induced toxicity has both short and long-term components as revealed by survival rate and oxidative stress measurements at 24 h (Figure 4, Figure 5B and 5C) and by lifespan data (Figure 5A). The protective effect afforded by SKN-1 upregulation upon Mn exposure and its nuclear relocation in ASI neurons (Figure 6) emphasizes the influence of acute Mn treatments on aging. ASI neurons which strongly express SKN-1, were shown to play a key role in lifespan modulation through the well-studied diet-restriction pathway [125]. Hence, further examination of Mn-induced toxicity and lifespan reduction in ASI neuron-ablated worms, *skn-1(RNAi)* worms and mutants of the diet-restriction pathway may provide essential insights in the relationship between aging and PD, as well as on the role of specific brain area (corresponding to the *C. elegans* ASI neurons) on aging and PD.

Despite the absence of an obvious PD-like behavior in the worm, DAergic neurodegeneration in *C. elegans* induced either by PD-drug treatment (6-OHDA, DA, MPP^+^) or by Mn involves the same key genes and molecular pathways as in vertebrates [32], [36], [54], [59], [60], [64], [65], [66], [126], [127], [128], has both short-term and long-term components and therefore represents a powerful model to investigate genetic and environmental causes of PD and manganism. To further demonstrate the relevance of our findings to vertebrate physiology, it would be interesting to test (1) whether co-treatments of DA and Mn lead to the same synergy in DAergic neurodegeneration in rodents; (2) whether such synergy is dependent on DAT; and (3) whether tyrosine hydroxylase (TH/CAT-2) and VMAT inhibition affords neuroprotection. Establishing the contribution of vertebrate NADPH dual-oxidases to the oxidation state of DA in the absence or presence of Mn would also be important. The present study also revealed DA-dependent mechanisms of Mn-induced toxicity by focusing on a whole-organism approach, while acknowledging that further investigation should also focus specifically on DAergic neuron physiology. For instance, determining if upon Mn exposure, *cat-2*, *cat-1* and *cat-2;dat-1* mutants display less or no neurodegeneration and if DA supplementation can reverse this effect, would allow clearer understanding of the role of DAT, and intracellular and extracellular DA in the demise of the DAergic system. It would also be important to develop full-length GFP-tagged protein markers of the DAergic neurons as the *dat-1::*GFP probe can only reveal relatively advanced stages of neuronal decay, and not more subtle functional changes. This point is exemplified by the observation of SKN-1::GFP relocation in ASI neurons (Figure 6), which would have been missed if only a transcriptional GFP reporter approach had been used. Finally, exploring the effect of aging and diet-restriction modulated by the ASI neurons and the Nrf2-like transcription factor, SKN-1, would enhance our understanding of the pathophysiology and the progressive nature of PD.

### Further developments and implications for the treatment of DAergic neurodegeneration

Given the similarities between Mn toxicity in *C. elegans* and in vertebrates [66] as well as the great conservation of most PD-related genes involved in DAergic neurodegeneration in rodents and humans [32], [36], [59], [60], [64], [129], it is likely that the DA-dependent toxicity revealed herein plays an important role in diseases associated with DAergic neuron loss in mammals. The importance of extracellular DA levels in the toxicity mechanisms described here, if corroborated in vertebrates, could bear important implications for the treatment of DAergic neurodegeneration, as modulating extracellular *vs.* intracellular DA levels requires different strategies. For instance, L-DOPA is prescribed in PD and manganism patients to treat the tremor and bradykinesia arising from the loss of DAergic activity. If L-DOPA, like DA, led to the excessive generation of oxidized reactive species *in vivo*, L-DOPA treatment, although compensating for the DA loss, could accelerate or exacerbate the DAergic neurodegeneration over a longer term. If the involvement of dual-oxidases in DA oxidation was confirmed in vertebrates and if L-DOPA was shown to be easily oxidized by dual-oxidases, it would be important to design alternative DA analogues, which maintain high affinity for DA receptors and DAT but are poor substrates for the dual-oxidases. Another strategy to limit the extent of the DAergic neurodegeneration could be the direct inhibition of the dual-oxidases. Finally, the protective effect of SKN-1 overexpression on Mn-induced toxicity (Figure 6A), which mirrors the neuroprotection afforded by astrocytic overexpression of Nrf2 in mice [130], suggests that promoting Nrf2 activity may be beneficial in limiting Mn-induced toxicity [131], [132]. Such an approach would be particularly relevant to welding and smelting activities in which workers exposed to metal fumes have an increased risk of developing parkinsonian syndromes [28], [133], [134], [135], [136].

### Nature of the DA-derived oxidized species and oxidative-stress pathways involved in PD

The current literature provides a conceptual framework for addressing the synergistic mechanism of Mn and DA in DAergic neurodegeneration [76], [137], [138], [139], [140], [141]. Accordingly, Mn may enter DAergic neurons via DAT as a complex with DA-derivatives, such as dopaminochrome [138], [141], [142], [143], [144], [145], [146], [147]. This hypothesis provides explanations both for the specificity of Mn toxicity towards DAergic neurons as well as for the synergistic toxicity of extracellular DA and Mn, while remaining consistent with studies that report a significant Mn uptake by the DAergic neurons [148], [149], [150], [151]. Purification and identification of the DA-derived reactive species (using HPLC-ED [152]) are essential for proving these suggestions. Further quantification of those DA-derived species in Mn-, MPP^+^-, 6-OHDA- and DA-exposed worms or in the rodent brain and cerebrospinal fluid should help identify the key toxic species in DAergic neurodegeneration as well as point out candidate enzymes possibly involved in PD. However, in biochemical approaches, the timing of extraction is critical to robustly detecting and measuring those species. This concern is exemplified by our oxidative stress marker measurements in wild-type and *dat-1(ok157)* mutant worms (Figure 5B and 5C). Hence, above the Mn dose reaching the maximum increase in ROS or F2IP levels, higher Mn doses led to lower oxidative stress marker levels suggesting a decrease in oxidative stress, while the lethality rate at 24 h and lifespan results suggest otherwise (Figure 4, Figure 5A). It is unlikely that oxidative stress decreased past the dose showing the maximum increase in oxidative stress markers. Downstream conversion or degradation of ROS or F2IP associated with a worsening of the condition of the worm may be responsible for this effect. Using genetic backgrounds that stop or delay the oxidative cascade at different steps of the pathway would allow the accumulation of specific ROS and increase their detection. Hence, Mn-resistant *C. elegans* mutants exposed to medium- to high-dose treatments (in the 20 to 100 mM range) could be very helpful in identifying the early steps of the oxidative cascade(s) leading to DAergic neurodegeneration and could yield new molecular targets for PD treatment. This study identifies a few of the potential candidates (*bli-3*, *SKN-1* overexpressing and maybe *cat-1* mutant worms, Figure 4 and Figure 6), but genetic screens to isolate new Mn-resistant mutants would provide powerful means for further investigation.

### Role of Mn and DA in *C. elegans* longevity

In the course of this study, we came across several indications of the influence of the DA-dependent and Mn-induced toxicity on *C. elegans* longevity, which is unlikely to be a direct consequence of DAergic neurodegeneration, but rather a concomitant effect (*dat-1(ok157)* mutants did not show DAergic neurodegeneration, whereas their lifespan decreased upon Mn exposure).

First, the fact that SKN-1 protects against Mn-induced toxicity and modifies its nuclear localization pattern upon Mn exposure in ASI neurons (Figure 6), provides a genetic link with the Insulin/IGF-1 and caloric restriction pathway known to be involved in the modulation of lifespan in *C. elegans* [97]. The nuclear relocation of SKN-1 in ASI neurons in a punctuate pattern is suggestive of binding of this transcription factor to specific chromosomal regions, likely corresponding to the loci of its downstream targets. Chromatine immunoprecipitation experiments could be used to identify those loci, while generation of transgenic worms expressing SKN-1::GFP and tagged sequences corresponding to candidate gene (for instance the *superoxide dismutase* genes) regulatory sequences would allow immuno-colocalization of SKN-1 and its targets. Nrf2/SKN-1 is known to be a key regulator of antioxidant response from man to worm [94], [99], and its impact on lifespan could as well be due to its ability to reduce oxidative stress naturally occurring with age, without involving caloric restriction. Given the impact of Mn exposure and DA metabolism on oxidative stress (Figure 5B and 5C), SKN-1 overexpression may improve Mn-exposed worm survival merely by compensating for the excessive ROS produced when extracellular DA levels are high and/or when Mn exposure reaches toxicity levels. Interestingly, the survival dose-response curve obtained with a non-null mutation (*ok2315*) in a mixed population of heterozygous and homozygous animals (Figure 6A) showed that even for very low doses of Mn (0.001 and 0.01 mM), a noticeable fraction of the worms (about 15–20%) died at 24 h post treatment compared to untreated animals, making it difficult to fit the experimental data with a simple sigmoid dose-response curve. As it is a mixed population, it is likely that mostly homozygous mutants died at those lowest doses, suggesting that their LD_50_ could be in the submillimolar range, making *skn-1(ok2315)* worms the most Mn-sensitive mutants tested so far. This requires direct confirmation, but it further supports an essential role for SKN-1 in regulating even slight changes in oxidative stress levels.

Second, our lifespan experiments showed that both *dat-1* and *cat-2* mutants are short-lived in control conditions (no published literature could be identified reporting on the lifespan of these mutants) compared to wild-type worms (Figure 5A). According to our ROS and isoprostane measurements (Figure 5B and 5C), *dat-1* mutants show increased oxidative stress, which is known to worsen with age; therefore explaining their shorter lifespan. Conversely, *cat-2* mutants seemed much less affected by Mn-induced oxidative stress, as Mn treatment did not lead to detectable increase in ROS and only led to a significant increase in isoprostane levels above 10 mM of Mn exposure. Moreover, high Mn exposure was able to rescue the shorter lifespan of *cat-2* mutants (Figure 5A). Mn is naturally required for the antioxidant activities of several enzymes, such as catalase and superoxide dismutase, which activities are critical in the aging brain [153], [154], [155]. Mn also has bactericidal and fungicidal [156] properties that are exploited in pesticides, such as Maneb. Several hypothesis can be formulated: (1) *cat-2* mutants may take up less Mn than wild-type worms, possibly below optimal levels, (2) DA depletion in *cat-2* mutants could make them feed improperly and somehow normally toxic Mn doses would restore the metabolic balance in those worms, (3) *cat-2* worms may be prone to infections and high Mn exposure would help the worms cope with a weak immunity. The first point could involve DA as a regulator of feeding behavior. This aspect can be tested by measuring Mn levels, the basal slowing response [157], sharp angle turns [158] and the pharyngeal pumping rate of *dat-1*, *cat-2*, *cat-2;dat-1* and wild-type animals upon Mn exposure. Those tests would also allow functional characterization of the DAergic circuit of Mn-exposed worms. The second hypothesis involves energy depletion as a cause of shortened lifespan, whereas the stress of Mn exposure would restrict energy expenditure by inhibiting the Insulin/IGF-1 pathway, possibly involving SKN-1 activation. In this case, Mn intake may not be different from other worms. Mn level measurements, Nile red and oil red O [159] staining to monitor fat stores, as well as RT-PCR and western-blots to measure *daf-2*, *akt-1/-2*, *sgk-1*, *daf-16* and *skn-1* expression levels would allow to test this idea. The third hypothesis implies that DA-derived ROS naturally play a role in the worm's immunity and that a high Mn dose compensates for their absence in *cat-2* mutants. The reasoning behind this idea is detailed in the next paragraph. This could be easily tested by comparing the resistance to infections of wild-type, *dat-1*, *cat-2*, *cat-2;dat-1* and *bli-3* mutants in presence or absence of Mn.

### Mn, DA, BLI-3: a link between immunity and neurodegeneration?

Our data show that DA and BLI-3-dependent ROS production is triggered or amplified by Mn, which was shown to be taken up by the NRAMP/DMT orthologues, *smf-1*, *-2* and *-3* [66]. Recently, *bli-3* and BLI-3-generated ROS have been implicated in *C. elegans* defense against bacteria and fungi [105], [106]. The NRAMP/DMT family of metal transporters has a well-established role in innate immunity in vertebrates [160], [161], [162], [163]. In *C. elegans*, NRAMP/DMT orthologue deletion mutants were found to be hypersensitive to *Staphyloccocus aureus* infection, which was rescued by Mn feeding [164]. Involvement of BLI-3-generated ROS, Mn and NRAMP/DMT in the defense against pathogens in *C. elegans*, as well as in DA-dependent neurodegeneration, raises the question of a link between immunity and DAergic neurodegeneration. The finding that DA and Mn act synergistically in a BLI-3-dependent ROS production pathway suggests that DA may play a direct role in the worm's immunity. If this mechanism is conserved in vertebrates, perhaps as a relic of a primitive immune system, it could bear interesting implications for brain physiology. For instance, DA-derived ROS could help fight infections when the blood-brain barrier (BBB) is compromised. On the other hand, they could also injure the brain and more specifically the DAT-expressing DAergic neurons. Infections and inflammation have long been suspected to play a role in the etiology of PD, as various infections have been associated with cases of PD [165], [166], [167], [168], [169], [170], [171], [172]. The identification of a DA-derived ROS production mechanism implicating dual oxidases may hold some clues for the understanding of those associations.

### Summary

This study confirms the conservation across the animal kingdom of molecular pathways involved in manganism and PD, provides the grounds for further biochemical and genetic investigations using the *C. elegans* model to tackle the complex issue of environment-gene interactions in age-related DAergic neurodegenerative disorders, and unravels the essential role of extracellular DA oxidation and the NADPH dual-oxidase, BLI-3, upstream of DAT-1 requirement in the neurodegeneration pathway. The data also point to a genetic link with distinct and more general aging processes, such as the diet-restriction pathway through the involvement of SKN-1, and with an innate immunity genetic network involving metal-content regulation via the SMF transporters and oxidative defence mechanisms via BLI-3 in *C. elegans*.

## Materials and Methods

### *C. elegans* strains and handling of the worms

*C. elegans* strains were handled and maintained at 20°C as previously described [173]. The following strains were used: N2 (+); BY200, *dat-1*::GFP*(vtIs1) V*; BZ555, *dat-1*::GFP*(egIs1)*; VH15, *glr-1*::GFP*(rhIS4) III*; LX929, *unc-17*::GFP*(vsIs48)*; EG1285, *lin-15B(n765) unc-47::GFP(oxIs12) X*; DA1240, *adIs1240[lin-15((+) eat-4*::GFP) *lin-15B(n765) X*; LX734, *dop-2(vs105) V*; *dop-1(vs100) dop-3(vs106) X*; LX831, *lin-15B(n765) X*; DOP-1::GFP*(vsIs28) dop-3::*RFP *(vsIs33)*; RM2702, *dat-1(ok157) III*; MT9772, *mod-5(n3314) I*; CB1111, *cat-1(e1111) X*; CB1112, *cat-2(e1112) II*; CB767, *bli-3(e767) I*; BY602, *cat-2(e1112) II*; *dat-1(ok157) III*; BY645, *dat-1(ok157) III*; *dat-1*::GFP*(vtIs1) V*; VC1772, *skn-1(ok2315) IV;nT1[qIs51](IV;V)*. All strains were provided by the Caenorhabditis Genetic Center (CGC, Minnesota), except for the BY602 and BY645 strains, which were generously provided by Randy Blakely (Vanderbilt University Medical Center, TN, USA).

### Acute manganese chloride treatments

Acute (30 min) manganese chloride (MnCl_2_) treatments were performed on 5,000 synchronized L1 per sample, and live worms were scored 24 h later, as previously described [66]. Scores were normalized to percent control (0 mM MnCl_2_ exposure).

### Acute dopamine treatments

Dopamine (Sigma Chemical Co., St Louis, MO) solutions were prepared in M9 buffer. Acute (30 min) treatments on young L1 worms were first tested from 1 mM to 50 mM DA, guiding us in choosing 10 mM as the working sub-lethal dose. Synchronized L1 were acutely pre-treated with 10 mM DA for 10 min, washed 5 times in 85 mM NaCl solution and subjected to MnCl_2_ acute treatments. Control worms were pre-treated with M9.

### Dopamine content measurement

Synchronized L1 worms were collected, washed three times in 85 mM NaCl and distributed in tetraplicates of 200,000 L1. Worms were pelleted and the supernatant was removed. The tubes were then immediately frozen in liquid nitrogen and stored at −80°C. For each tube, the worm pellet was re-suspended in lysis buffer containing EDTA to scavenge free metal ions and was sonicated to disrupt cell membranes. Fifty µL of the lysate was used to perform a BCA assay to measure protein levels. Isoproterenol was added as an internal standard to the remaining 250 µL of the lysate, which was applied to the aluminum membrane to bind DA. Collected DA samples were then processed for High Performance Liquid Chromatography (HPLC). To correct for inter-sample variations in the extraction efficiency, the ratio of DA to the internal standard, isoproternol, was estimated, and the total DA content was calculated relative either to protein levels or to the number of worms.

### Isoprostane content measurement

Worms were grown at high densities on 8P-plates. 20,000 synchronized L1 per sample were washed off the plates in 85 mM NaCl, collected in 10 mL 85 mM NaCl and acutely treated (30 min under gentle agitation) with MnCl_2_. Worms were pelleted and washed three times in 85 mM NaCl and then re-suspended in 85 mM NaCl, 0.5% Triton X-100, 5 mM Tris Buffer pH 6.8, 0.5× protease inhibitor cocktail (Sigma P8340) with zirconia beads, up to 1 mL. Samples were then processed with a Mini beadbeater-16 (Biospec Products, OK, USA) for 7 cycles of 20 s and kept 1 min in ice-cold water after each cycle. 20 µL of supernatant per sample were kept for measurement of protein levels by the Bradford method. 850 µL were added to 10 mL Folch solution and gently shaken every 5 min for 30 min. 2 mL of 0.9% NaCl per tube were added. Tubes were centrifuged at 3,000 rpm for 10 min at 4°C, the aqueous layer was discarded, and the organic phase was dried under nitrogen flow at 37°C. The detailed procedures for the purification and derivatization steps were previously described [174].

### ROS measurement

Synchronized L1 were acutely treated with MnCl_2_ as described earlier and washed 4 additional times in M9 buffer. 2′7′ dichlorodihydrofluorescein diacetate (H2DCF-DA) was added at 1 mM for one hour in the dark. Worms were then washed 4 times in M9 buffer. Worms were frozen and thawed twice and homogenized by sonication and then centrifuged. The supernatants were transferred to a 96-well plate and their fluorescence levels (excitation: 485 nm; emission: 535 nm) were detected using a FLEXstation III (Molecular Devices, Sunnyvale, California) pre-heated at 37°C. The fluorescence from each well was measured every 20 min for up to 2 h. Here, we report values obtained at 1 h. Fluorescence measurements were normalized to time zero values, and rates of increase in fluorescence (reflecting ROS levels) were expressed as percent control. Measurements were repeated 3 times, each condition was performed in triplicate, and the experiment was repeated in three independent worm preparations for each tested strain.

### Lifespan experiments

Synchronized L1 worms were acutely exposed to MnCl_2_ concentrations as described earlier. Live and healthy-looking worms (60–66 per condition) were collected on the same day at the late L4 stage and transferred every five days to new OP50-seeded NGM plates. Survival was assessed every two to three days until all worms had died. All tested *C. elegans* strains were assessed in parallel, and each experiment was performed three times, yielding qualitatively identical results. Plotted curves represent averages of those triplicate independent experiments.

### Epifluorescence, DIC, and confocal microscopy

For each slide, at least 30 worms were mounted on 4% agarose pads in M9 and anaesthetized with 0.2% tricaine/0.02% tetramisole in M9. Fluorescence observations and scoring of neuronal defects were performed with an epifluorescence microscope (Nikon Eclipse 80i, Nikon Corporation, Tokyo, Japan) equipped with a Lambda LS Xenon lamp (Sutter Instrument Company) and Nikon Plan Fluor 20× dry and Nikon Plan Apo 60× 1.3 oil objectives. Confocal images acquired for illustration or GFP intensity measurement purposes were captured through Plan-Neofluar 40×, Plan-Apochromat 63×, or Plan-Neofluar 100× oil objectives with a 1.3, 1.4 and 1.3 apertures, respectively, on a LSM510 confocal microscope (Carl Zeiss MicroImaging, Inc.) scanning every 200 nm for XZ sections. Images were processed with the Zeiss LSM Image Browser 4.0.0.157 software and edited using Photoshop 7.0 (Adobe). Microscopes were housed in air-conditioned rooms (20–22°C). Amphid and phasmid neuron staining was performed following MnCl_2_ acute treatment by soaking the worms for 2 h in a 10 µg/mL DiI solution prepared with M9, washing off the dye for 1 h in M9 and then recovering the worms on OP50 coated NGM plates.

### SKN-1::GFP fluorescence measurements

SKN-1::GFP transgenic worms were acutely treated as previously described, transferred to OP50-1 seeded NGM plates and imaged 1 h post-treatment. Fluorescence measurements of the SKN-1::GFP signal were performed on complete confocal Z-stack maximal projections of L1 *C. elegans* ASI neurons. Treated and untreated animals were mounted on the same slide and imaged with the same magnification, gain, offset, pinhole and laser power settings. GFP integral intensity and signal density of the maximal projection of the ASI nuclei were measured with the freeware ImageJ (developed by Wayne Rasband, NIMH, Maryland, USA).

### Statistics

Dose-response lethality curves, longevity curves and histograms for DA, isoprostane or ROS content measurements were generated with GraphPad Prism (GraphPad Software Inc.). We used a sigmoidal dose-response model with a top constraint at 100% to draw the curves and determine the LD_50_ or the average lifespan values reported in the graphs. Statistical analysis of significance was carried out by one-way ANOVA for the dose-response curves, longevity curves and dopamine measurements; two-way ANOVA was used to measure isoprostane and ROS content, followed by post-hoc Bonferroni test when the overall p value was less than 0.05. For SKN-1::GFP fluorescence analysis, unpaired two-tailed T-test was used to assess statistical differences in mean values. In all figures, error bars represent SEM; * refers to differences between genotypes; # refers to differences between doses; */# p<0.05; **/## p<0.01; and ***/### p<0.001.

## Supporting Information

Figure S1 *mod-5* loss of function does not affect Mn-induced lethality. The serotonin re-uptake transporter (SERT) mutant *mod-5(n3314)* is not significantly different from wild type (p>0.05) regarding Mn sensitivity, and is characterized by an LD50 = 45 mM.
(0.12 MB TIF)

Figure S2 *cat-2(e1112)* exhibits higher ROS levels than wild type upon Fe exposure. Both wild-type and *cat-1(e1112)* worms display significantly higher ROS levels upon 1 mM FeSO4 acute exposure (p<0.01). *cat-1(e1112)* mutants also show a significant increase compared to wild-type worms (p<0.05).
(0.10 MB TIF)
