# Supplementary figures and images for: Extracellular Dopamine Potentiates Mn-Induced Oxidative Stress, Lifespan Reduction, and Dopaminergic Neurodegeneration in a BLI-3–Dependent Manner in Caenorhabditis elegans
Source: PLoS Genet. 2010 Aug 26;6(8):e1001084. doi: 10.1371/journal.pgen.1001084 (PMC2928785; doi:10.1371/journal.pgen.1001084)

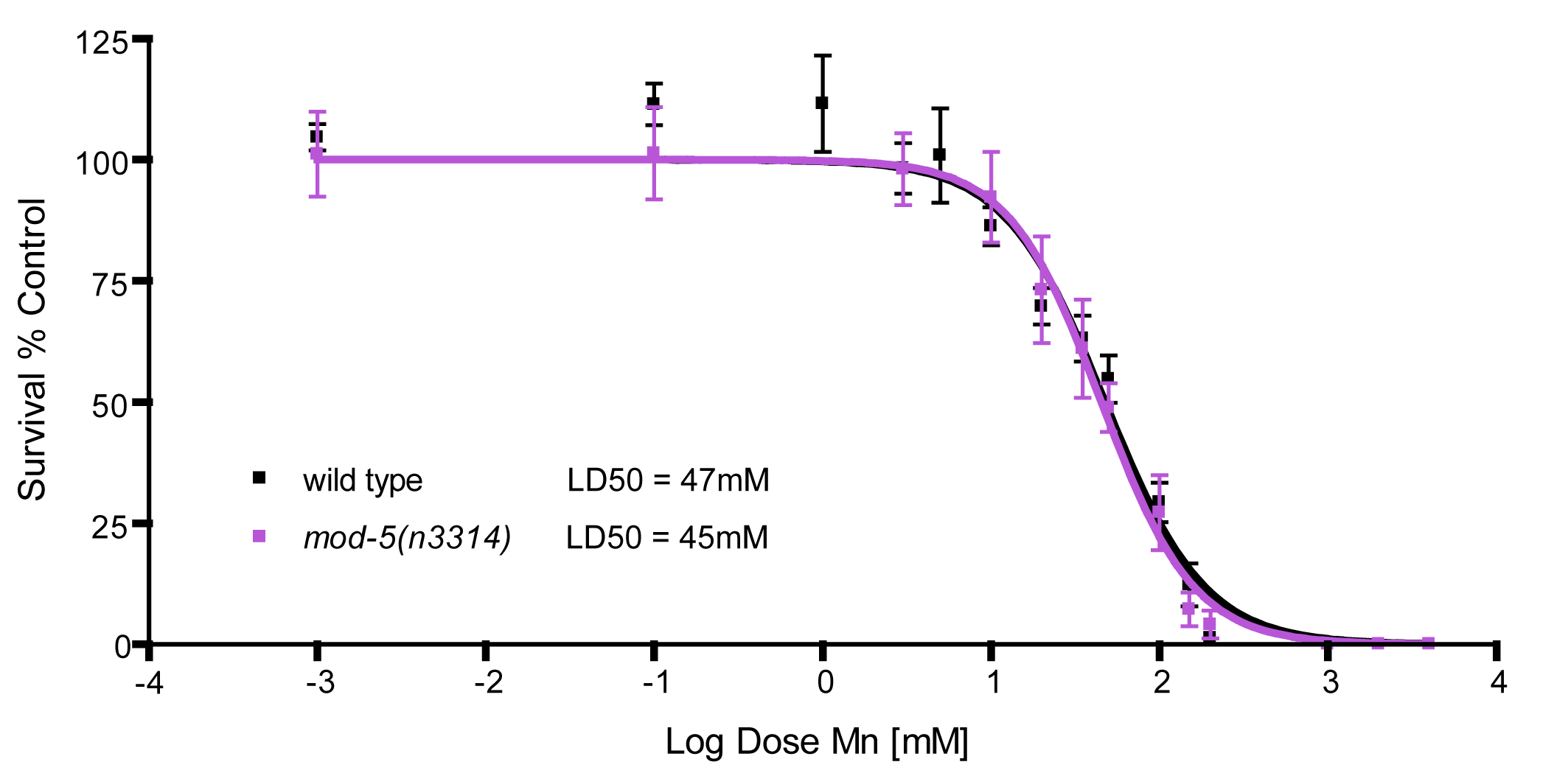

Supplement: Figure S1 — mod-5 loss of function does not affect Mn-induced lethality. The serotonin re-uptake transporter (SERT) mutant mod-5(n3314) is not significantly different from wild type (p>0.05) regarding Mn sensitivity, and is characterized by an LD50 = 45 mM. (0.12 MB TIF) [file pgen.1001084.s001.tif]

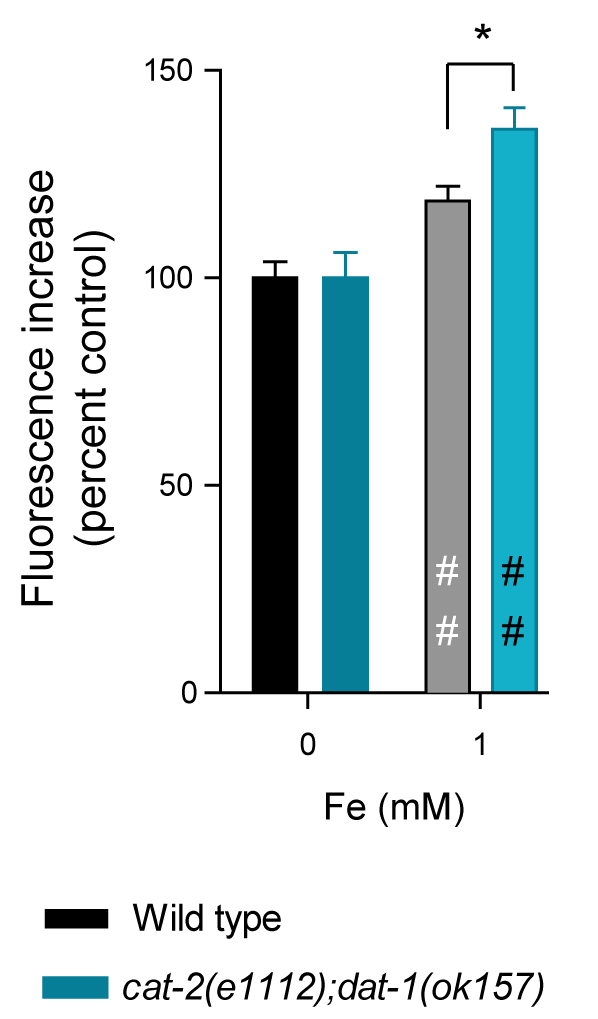

Supplement: Figure S2 — cat-2(e1112) exhibits higher ROS levels than wild type upon Fe exposure. Both wild-type and cat-1(e1112) worms display significantly higher ROS levels upon 1 mM FeSO4 acute exposure (p<0.01). cat-1(e1112) mutants also show a significant increase compared to wild-type worms (p<0.05). (0.10 MB TIF) [file pgen.1001084.s002.tif]
